# Preoperative pancreaticobiliary stenting: A refined technique for safe and successful duodenal papillectomy

**DOI:** 10.1055/a-2930-8908

**Published:** 2026-08-19

**Authors:** Manling Lang, Bin Yang, Mingwen Guo

**Affiliations:** 1 The Second Affiliated Hospital of Soochow UniversitySuzhouJiangsuChina; 2 Department of GastroenterologyQionglai Medical Center HospitalQionglaiSichuanChina

Papillectomy is a technically demanding procedure performed during endoscopic retrograde cholangiopancreatography. After resection, pancreatic and biliary stents are routinely placed to prevent complications such as pancreatitis, delayed perforation, and hemorrhage. However, cannulation of the bile and pancreatic ducts becomes difficult after the papilla has been removed, posing a major clinical challenge. To resolve this issue, we developed an innovative technique of preoperative stent placement before papillectomy.

**Fig. 1:**
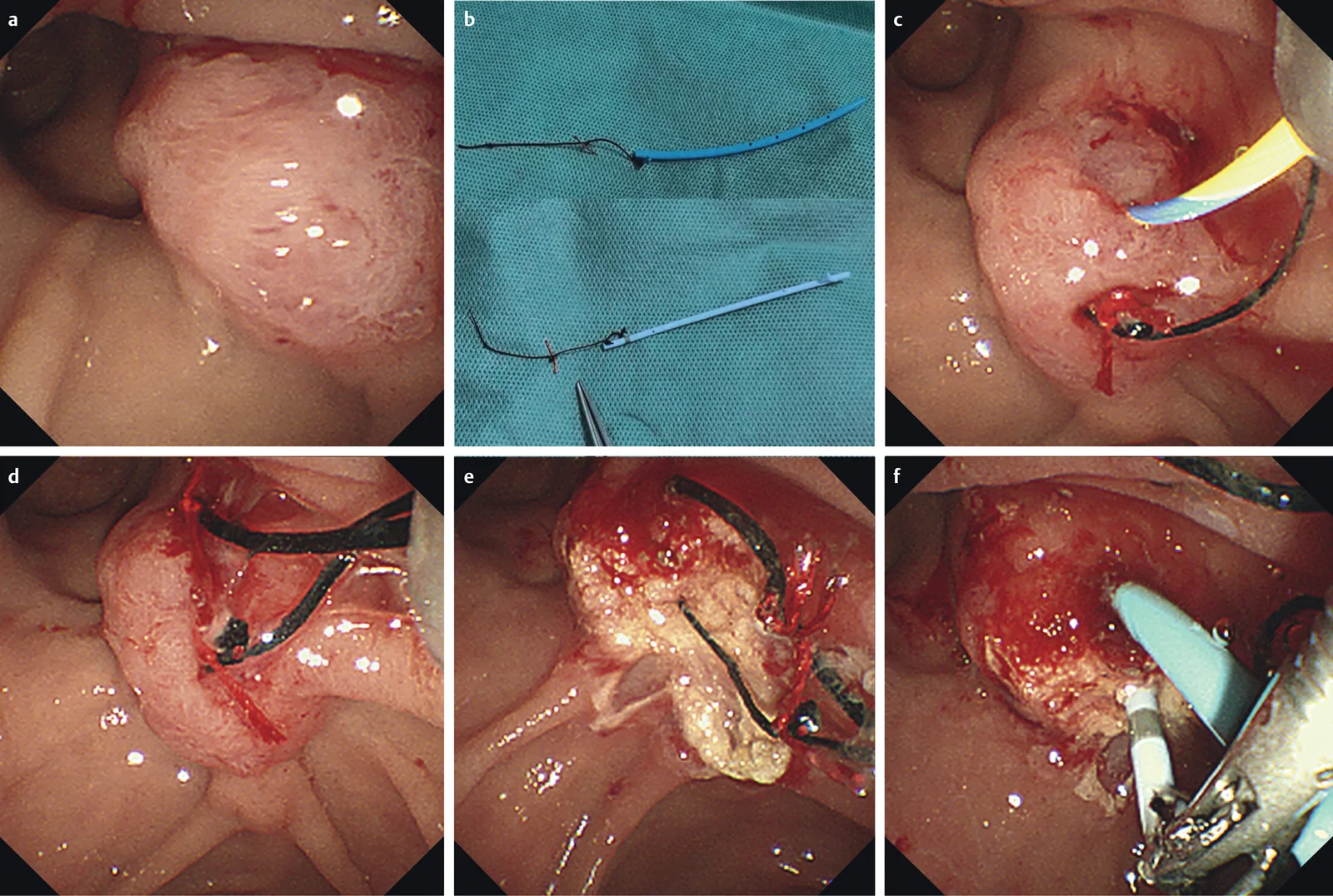
An innovative papillectomy strategy: preoperative pancreaticobiliary stenting guarantees safety and operative success. ( **a** ) Adenomatous changes of the duodenal papilla on white-light endoscopy. ( **b** ) Modified biliary (7 Fr, 5 cm) and pancreatic (5 Fr, 5 cm) stents were fitted with 4-cm No. 0 surgical sutures secured at their distal extremities; the red marking thread serves as markers for the insertion depth. ( **c** ) The pancreatic stent is fully deployed in the pancreatic duct, with the red marker suture left external to the papilla. ( **d** ) The biliary stent is fully deployed in the bile duct, with the red marker suture left external to the papilla. ( **e** ) Endoscopic papillectomy completed, with all sutures remaining in situ. ( **f** ) The biliary and pancreatic stents are pulled outward through sutures using biopsy forceps.

A 78-year-old man was diagnosed with duodenal papillary adenoma by endoscopy and biopsy at an outside institution. He was admitted to our hospital for endoscopic papillectomy. White-light endoscopy demonstrated adenomatous changes of the duodenal papilla ( Fig. 1a ). We modified both stents by tying a 4‑cm No. 0 surgical suture to the distal end of each: the biliary stent (7 Fr, 5 cm) and the pancreatic stent (5 Fr, 5 cm), and used a red ordinary thread to mark the insertion depth ( Fig. 1b ). The pancreatic stent was fully deployed into the pancreatic duct, leaving the red marking suture external to the papilla ( Fig. 1c ). Similarly, the biliary stent was fully deployed into the bile duct, with its red marking suture left outside the papilla ( Fig. 1d ). Endoscopic resection of the duodenal papilla was performed ( Video 1 ), and all sutures remained in situ after resection ( Fig. 1e ). The resected ampullary specimen was disengaged from the suture using a snare and placed in the gastric cavity ( Video 1 ). Biopsy forceps were used to pull the biliary and pancreatic stents outward via the pre‑attached sutures, thereby exposing the stents over the post‑resection defect to drain bile and pancreatic juice ( Fig. 1f ). Finally, the resection defect was closed with through‑the‑scope clips ( Video 1 ).

**Video 1** This video demonstrates a refined preoperative pancreaticobiliary stenting technique for safe endoscopic duodenal papillectomy. Endoscopic examination reveals adenomatous changes of the duodenal papilla. Prepare biliary and pancreatic stents with sutures tied at the distal end. Red sutures mark the insertion depth. Selective pancreatic duct cannulation. Guidewire enters the pancreatic duct. Fully deploy the pancreatic stent inside the duct. The red suture marks the insertion depth. Selective biliary cannulation. Guidewire enters the common bile duct. Fully position the biliary stent inside the duct. The red suture marks the insertion depth. Snare the duodenal papilla. Electrosect the duodenal papilla. Following ampullary electroresection, the snare was withdrawn. The resected ampullary tissue was retrieved from the suture using a snare. The resected ampullary specimen positioned in the gastric cavity. Use biopsy forceps to pull out the pancreatic stent by its suture. Use biopsy forceps to pull out the biliary stent by its suture. Close the wound with tissue clips.


Successful selective cannulation of the biliary and pancreatic ducts remains unpredictable after papillectomy, which represents a major technical hurdle of this procedure. Our novel technique relies on preoperative pancreaticobiliary stenting. After resection, the stents are pulled through the resection wound via pre-attached sutures to maintain the effective drainage of bile and pancreatic secretions and substantially reduce the risk of postoperative complications. This approach completely bypasses the aforementioned technical difficulty. It is especially suitable for less experienced endoscopists and deserves widespread clinical applications.

Endoscopy_UCTN_Code_TTT_1AR_2AD

Endoscopy_UCTN_Code_TTT_1AR_2AZ

